# Barriers and facilitators to HIV testing among African and Caribbean heritage communities: a mixed methods study

**DOI:** 10.1136/sextrans-2025-056491

**Published:** 2025-05-13

**Authors:** Temilola Adeniyi, Jeremy Horwood, Marsha Doran, Khabo Piggott, Aisha-Monic Namurach, Lindsey Harryman, Emmy Oldenbourg, Miryam Kiflu, Nathan Speare, Mary Griffin, Matthew Wilson, Mark Febrache, Rachel Allbless, David Dravie-John, Joanna Copping, Frank De Vocht, Scott Walter, Fiona Fox

**Affiliations:** 1 Population Health Sciences, University of Bristol, Bristol, UK; 2 University of Bristol Centre for Academic Primary Care, Bristol, UK; 3 NIHR Applied Research Collaboration West, Bristol, England, UK; 4 Brigstowe Charity, Bristol, UK; 5 University Hospitals NHS Foundation Trust, University Hospitals Bristol and Weston NHS Foundation Trust, Bristol, UK; 6 Brook, Bristol, UK; 7 Terrence Higgins Trust, London, UK; 8 Bristol Health Partners, Academic Health Science Centre, Bristol, UK; 9 African Voices Forum Ltd, Bristol, UK; 10 Bristol City Council, Bristol, UK

**Keywords:** HIV, Ethnic Groups, HIV Infections, Pre-Exposure Prophylaxis, SEXUAL HEALTH

## Abstract

**Abstract:**

**Objectives:**

African and Caribbean heritage (ACH) communities in the UK face disproportionately high rates of HIV and often experience delayed diagnoses, worsening health inequities. Increasing HIV testing in these communities is essential to address these disparities and support the UK’s HIV reduction targets. This study examines barriers and facilitators to HIV testing among Bristol’s ACH community, a high-prevalence area with significant rates of late diagnoses, filling a critical gap in context-specific data.

**Methods:**

Using a mixed-methods approach, this study combined 29 in-depth interviews and 41 online surveys, capturing ACH community members’ views on HIV stigma, healthcare trust and testing experiences. Data were thematically analysed and mapped to the Social Ecological Model (SEM) framework, with community researchers conducting data collection and analysis to enhance participants’ engagement and trust and contribute to a deeper contextual analytical understanding.

**Results:**

Findings highlight significant barriers across SEM levels: individual-level knowledge gaps and stigma, interpersonal confidentiality concerns within tight knit communities, community-level taboos and distrust and organisational barriers, such as discriminatory healthcare experiences. Effective facilitators included culturally specific services, flexible testing options, community-driven outreach and increased healthcare representation, all of which fostered greater trust and engagement in testing.

**Conclusion:**

The study underscores the importance of culturally aligned interventions, including representation within and training in cultural competence for healthcare providers and community co-production in service design. Implementing such strategies could reduce late diagnoses and support the normalisation of routine HIV testing in ACH communities, ultimately contributing to health equity. Future research should explore gender and age-specific barriers, while assessing the long-term impact of community-led interventions to inform national HIV policy and public health strategies for marginalised communities in the UK.

**WHAT IS ALREADY KNOWN ON THIS TOPIC**

- African and Caribbean heritage (ACH) communities are disproportionately affected by HIV in the UK.
- Barriers to HIV testing in these communities include stigma, cultural barriers and systemic inequities.
- Limited research has focused specifically on barriers and facilitators to HIV testing among ACH communities in the UK.

**WHAT THIS STUDY ADDS**

- Provides context-specific insights into HIV testing barriers and facilitators for ACH communities, a high-prevalence area with significant late diagnosis rates.
- Reveals persistent knowledge gaps about modern HIV prevention and treatment methods, even when basic understanding of transmission is good.
- Highlights the potential of culturally aligned services and community outreach in overcoming organisational and community-level barriers.
- Demonstrates the interplay between different levels of the Social Ecological Model in shaping HIV testing behaviours.

Reveals specific barriers to HIV testing in ACH communities, including:

- Fear of being seen at clinics by other community members due to closely connected local social networks.
- Persistent misconceptions about HIV as a ‘death sentence’ despite advances in treatment.
- Challenges in accessing services, such as difficulty getting appointments.
- Unclear instructions for self-sampling kits.

Identifies effective facilitators for HIV testing, such as:

- Community-based outreach in trusted spaces like barbershops.
- Dedicated ACH clinics that provide culturally safe environments.
- Normalising testing as part of routine healthcare rather than associating it only with illness.

**HOW THIS STUDY MIGHT AFFECT RESEARCH, PRACTICE OR POLICY**

- Informs the development of targeted, multilevel interventions to increase HIV testing uptake among ACH communities.
- Further demonstrates the need for the implementation of culturally tailored services and community-based outreach programmes.
- Emphasises the need for in-depth cultural competence training for healthcare providers.
- Emphasises the need for diversification of clinical staff.
- Highlights the importance of co-producing service improvements with community members.
- Suggests directions for future research, including exploring age and gender differences in testing barriers and evaluating long-term impacts of culturally tailored interventions.

## Background

 The WHO has set a target to eliminate all new HIV transmissions by 2030.^1^ However, HIV continues to disproportionately affect marginalised groups,^2^ including people of African and Caribbean heritage (ACH).^3 4^ The UK government reports that there is an urgent need to improve access to, and uptake of HIV testing, especially among women who face elevated risks.^5^ Nationally, an estimated 2.3% of black African heterosexual men and 3.4% of black African heterosexual women are living with HIV in England contrasting starkly with the 0.02% prevalence in the overall adult heterosexual population.^6 7^ An estimated 5% of black African men and 6% of black African women living with HIV in England are unaware of their HIV-positive status.^7^

Health inequalities among ACH people stem from social and structural inequities. These inequalities may include language, cultural factors and racism, contributing to barriers which may limit access to sexual health services.^8^ These are exacerbated by UK ‘hostile environment’ policies, which may make ACH communities reluctant to access services.^9^ HIV stigma can lead to lower uptake of sexual health services,^10^ refusal of HIV testing, non-disclosure to partners and poor engagement in prevention approaches.^11^ These are further exacerbated by pre-existing inequities and intersection with a marginalised-group identity, for example, gender, socioeconomic status and sexual orientation.^12 13^

In recent years, significant advancements have been made in HIV prevention and treatment. These include pre-exposure prophylaxis (PrEP) for HIV prevention and Undetectable=Untransmittable (U=U), which means people with HIV who maintain an undetectable viral load cannot sexually transmit the virus to others.^14^ Despite these developments, awareness and understanding of these advances remain limited in many communities.^15^

Bristol is a high-prevalence HIV area with almost 1000 people living with HIV. In contrast to other Core Cities, a network of major cities in the UK outside of London, are collectively focused on driving economic and social development.^16^ Bristol shows the second-highest incidence of late HIV diagnosis.^10 17^ It is estimated that around 80 people in Bristol are currently unaware of their HIV diagnosis, which increases the risk of onward transmission.^10^ In 2021, 20% of new HIV diagnoses in Bristol were from ACH communities,^17^ despite these groups making up only 4% of the city’s population.^10^ To meet the WHO goal, Bristol became an HIV Fast Track City in 2019.^18^ Local collaborators were awarded a Health Foundation grant for their Common Ambition Bristol programme (CAB) aimed at developing co-production partnerships to tackle health inequities.^19^ CAB involves ACH community members working in equal partnership with sexual health staff, to co-produce interventions to reduce HIV stigma, increase HIV testing and improve wider engagement with sexual health services.^20^ The CAB interventions include community outreach in black-owned businesses, dedicated sexual health testing clinics and targeted health promotion events.

This paper reports data that were collected during an evaluation of the CAB interventions, which aimed to understand factors influencing HIV testing in Bristol’s ACH communities. Given limited previous research focusing on the barriers and facilitators to HIV testing among people of ACH in the UK,^2 4 21 22^ the current findings may be useful to inform the development of targeted interventions to increase testing rates.

## Methods

### Study design

A mixed methods study involving interviews and surveys.^23^ Recruitment and data collection were conducted by a team of community researchers (MD, TA, KP and WA) who are all African or Caribbean heritage and received training and supervision from the university researchers (FF and JH). The community researchers delivered surveys and conducted interviews with members of the ACH community who had experienced a CAB intervention.

### Recruitment

Online survey: community researchers recruited participants for the online survey at CAB targeted health promotion events and during community outreach visits. They shared a link to the online survey, which participants could complete on their smartphones, or on a study iPad.

Interviews: participants were recruited for interviews in two ways:

1. At the end of the online survey, participants could indicate if they were willing to be contacted to discuss taking part in an interview.
2. The community researchers approached potential participants at CAB-targeted health promotion events and during community outreach visits.

Participants providing contact details were emailed the information sheet and consent form. Interviews were scheduled by MD on consent. Eligible participants were ACH individuals aged 18 or older, purposively sampled to ensure diversity in age, gender, ethnicity and socioeconomic status.

### Data collection

Survey data were collected between November 2022 and November 2023. Participants received a £5 voucher if completed at the time, or entry into a £100 prize draw if completed later. Interviews were conducted from March to November 2023 via telephone, videocall or in person. Community researchers conducted interviews using a semi-structured guide, piloted and refined by FF and the team. The interviews lasted 30–60 min, were audio-recorded with consent and professionally transcribed. Participants received a £20 gift voucher for their time.

Both the survey and the interview topic guides covered HIV knowledge and stigma, experiences of HIV testing, local sexual health services and CAB interventions (see online supplemental materials).

### Analysis

#### Qualitative

Interview transcripts were anonymised and analysed using reflexive thematic analysis. TA initially coded all transcripts in NVivo V.12, then collaborated with MD and FF in an iterative review, to develop and refine a codebook of themes and subthemes. TA applied the final codebook to all transcripts, with MD and FF reviewing data to ensure coding consistency and accuracy. The Social Ecological Model (SEM), which provides a framework to understand the multilevel factors influencing health behaviours and outcomes,^24 25^ was used to further develop the themes. The SEM suggests that health behaviours are shaped by factors across individual, interpersonal, organisational, community and policy levels. This approach has been successfully applied in previous studies to understand multilevel influences on HIV testing and care.^26^,^28^

#### Quantitative

Survey results were summarised using counts and proportions. Following analysis of both data sets, the quantitative data was mapped onto the qualitative themes by FF and SW.

## Results

### The interview sample

Interviews were conducted with 29 participants, of whom 16 (55%) were men and 13 (45%) were women. The age of interviewees ranged from 19 to 56 years, with a median age of 37 years. Among men, the age range was 21–47, and among women, 19–56. Nine participants (31%) were of African heritage, 18 (62%) of Caribbean heritage and 1 (3%) of Asian heritage. 13 (45%) were educated to degree level.

### The survey sample

40 ACH-identified participants completed the survey. Of these, 27 (68%) identified as male and 13 (32%) as female. In terms of ethnicity, 24 (59%) were of African heritage, 14 (34%) of Caribbean heritage and 3 (7%) identified as white. 33 participants (83%) described their sexual orientation as heterosexual or straight, 2 (5%) as men who have sex with men and 5 (13%) preferred not to disclose. Ages ranged from 20 to 69 years with a median of 44. 25 participants (61%) reported having a degree-level qualification. 32 (80.0%) reported having previously tested for HIV, while 8 (20.0%) stated they had never been tested.

**Figure 1:**
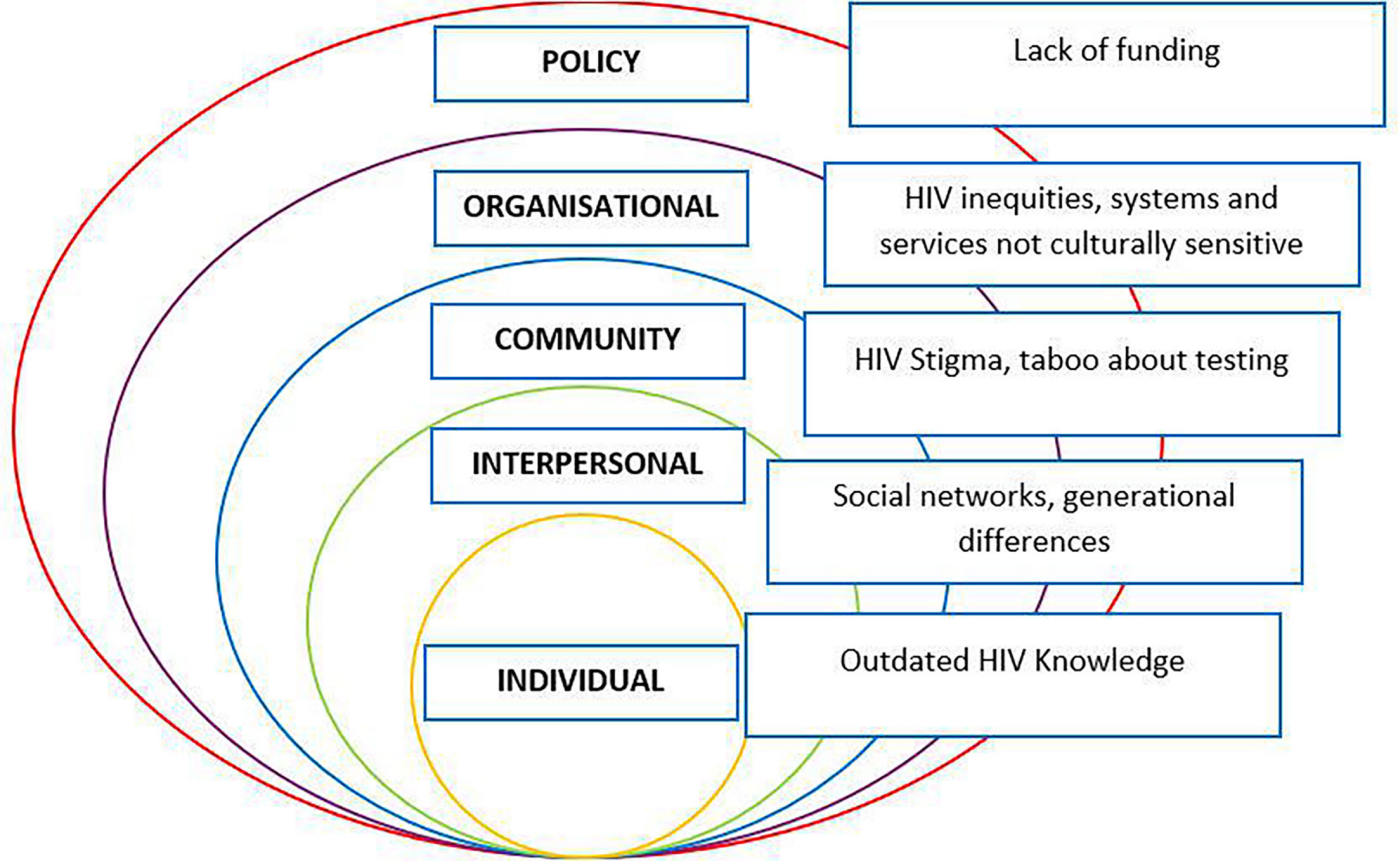
Barriers to HIV testing mapped on the Social Ecological Model.

The quantitative survey and qualitative interview data were amalgamated under the themes identified within the qualitative analysis, with demographics data summarised in table 1. They are presented under the SEM domains, to identify the multifaceted influences on HIV testing behaviour. Barriers and facilitators to HIV testing are presented within each level of SEM (individual, interpersonal, community, organisational and policy) in figures1  2 and are illustrated by interview quotations in tables2  3.

**Table 1:** Summary demographics characteristics for both interview and survey participants

Variable	Interview sample (n=29)	Survey sample (n=40)
Gender	55% men, 45% women	68% men, 32% women
Age range	19–56	20–69
Median age	37	44
Ethnicity	31% African, 62% Caribbean	59% African, 34% Caribbean, 7% white
Degree-level education	45%	61%
Ever tested for HIV	—	80%
Never tested for HIV	—	20%

**Figure 2:**
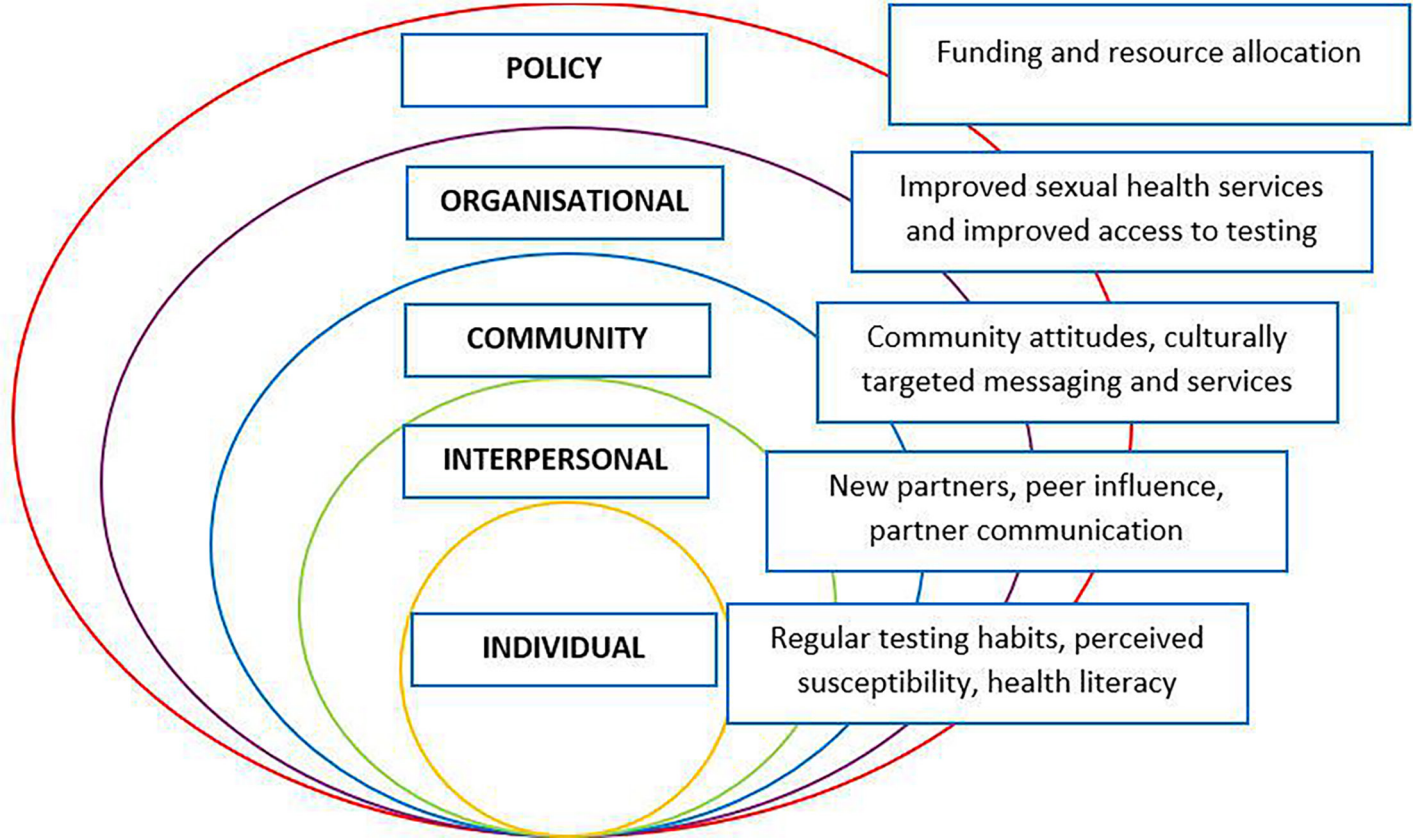
Facilitators to HIV testing mapped on the Social Ecological Model.

**Table 2:** Barriers to HIV testing: interview quotations

SEM levels	Barriers	Quote
Individual level	HIV knowledge	“I don’t know much about how it can pass but can it be holding hand or kissing?.” (Female, 33, Caribbean heritage) *“*I think the main thing is that you could just easily get it from them, like it’s COVID*.*” (Male, 21, African heritage)
Interpersonal level	Social networks	“I would just say it’s stigma, that’s what I think. They don’t want to be caught in the clinic when they first like oh what you are doing here, oh testing for HIV, you know all that embarrassment and stuff.” (Male, 21, African heritage)
	Generational differences	“I know somebody who died of HIV, she was an older neighbour of ours because she was too scared to ask for help, ask for treatment, just like silence ……I do think there are people who feel even if they do have HIV and even if they get treated, they won’t be able to mention it to their friend because of the embarrassment.” (Male, 21, African heritage)
Community level	HIV stigma	“there’s a lot that we don’t know, in the African Caribbean community and it’s also a taboo subject speaking about these things … it can be a well-hidden, you could say a taboo subject in terms of talking about*,* you know, sexual health in our communities.” (Male, 41, Caribbean heritage) “I’m pretty sure tomorrow if I told a friend that I’ve got HIV their attitude would change towards me drastically because they just don’t know that HIV is still out there like that.” (Male, 38, Caribbean heritage)
	HIV taboo about testing	“It’s almost like a dirty thing, isn't it? To go and get tested. I had to make up excuses just because I wanted that, but I shouldn’t have to lie about why I wanted it.*”* (Female, 56, Caribbean heritage) “Most people don’t want to do tests, from my own findings …. from my friends is people don’t want to do tests, because they don’t want to know their status. They’re scared to know their status.” (Male, 40, African heritage)
Organisational level	HIV testing inequities	“I think … black people’s access to healthcare is pretty poor, even in this country … they might fear that the doctors don’t know how to treat a black body …I know a lot of black people who’ve been to services and they’re like, ‘Oh, they didn’t even know where to put the needle in my arm. I came home stabbed 14 times by the nurse,’ things Like that.” (Female, 23 Caribbean heritage) “Because I’m African it’s like, you just think… I don’t know. Maybe I’m thinking too much about it, but I just feel like …I’m being really judged.” (Male, 35, African heritage) “I think you have to also factor in how the Black community has been let down by the NHS*.*” (Female, 56, Caribbean heritage)
Systems and services	Systems and services	“I mean, I really struggled. I called to book an appointment because I called the first day after 8:00, and they told me all the appointments are gone. Then they asked me to call, I think 8:00, or 8:30 the second day. I did call at that same time, exactly one min before, and I had to wait on the call for 50 minutes to get through to someone to talk to me.” (Male, 40, African heritage) “I shouldn’t have to go through all the hurdles to get tested, it should be easy.” (Female, 38, African heritage)

SEM, Social Ecological Model.

**Table 3:** Facilitators to HIV testing: interview quotations

SEM levels	Facilitators	Quote
Individual level	Personal motivators	“I had an auntie that passed away with AIDS. So, I also think my culture is … very aware. So, I know that it’s so close to me that I’ve always been tested regardless. So, me being in a marriage we still do those kind of things.” (Male, 35, African heritage) “I got tested because I had a new girlfriend.” (Male, 35, African heritage) “I aim … to make testing a habit every 6 months.” (Male, 41, Caribbean heritage)
Community level	Community attitudes	“I just feel like they (people living with HIV) should have a voice because that experience, it’s so good for people to know and to learn that they are still people and regardless of the fact that they have HIV, it’s just like any other disease out there really but I think normalising the conversation brings a balance.” (Male, 35, African heritage) “People to just keep talking about it (HIV). That’s just it. It’ll become very normal soon and there’s nothing to be embarrassed about.” (Female, 21, Caribbean heritage)
	Culturally targeted services and messaging	“I understand that it’s usually African Caribbean people that don’t want to go and get tested… So, I feel like it’s good that we have something where we can be surrounded by people like us and I suppose more comfortable [CAB ACH community clinic).” (Male, 40, African heritage) “This information that Common Ambition is doing is great. Now they’re coming to the people … Lots of Black people didn’t know about these PrEP or all these medications. Common Ambition is an improvement because at least they’re going out and letting people know, you know, letting us know that you can access this medication. You can access this information. As I said, I’ve never really gone to the local doctor out there or whatever that much, if at all to be honest. So, I think like this.” (Male, 41, Caribbean heritage)
Organisational level	Improving sexual health services	“The staff need more training on how to treat Black people.” (Female, 34, Caribbean heritage) “If they could see people like them or even on the literature, people on the leaflets that look like them.” (Female, 40, Caribbean heritage) “I think cultural competence is really important for these organisations…having some sort of sensitivity training. That would be important*.*” (Male, 35, African heritage)
	Improved access to testing	“Putting testing services or information at the back of a hair salon or nail salon, so not in the obvious places.” (Female, 19, Caribbean heritage) “That (information about how to book a test) should also be passed on as well because I thought the only way was to call. If they know that they can go online and get an appointment easily, because that was so easy, then that would get more people to come out and do the test.” (Female, 38, African heritage) “I can test myself at home without anyone knowing*.*” (Female, 34, Caribbean heritage)
Policy level	Funding and resource allocation	“More funding needs to be put into testing so it can reach more people especially who don’t go to the clinics.*”* (Male, 22, African heritage)

SEM, Social Ecological Model.

### Theme 1: barriers to HIV testing

#### Individual level

##### HIV knowledge

Interview participants noted significant HIV knowledge gaps in ACH communities, particularly around transmission, treatment options and testing benefits. Outdated views of HIV as a ‘death sentence’ and lack of awareness of scientific advances in prevention and treatment contributed to avoidance of testing or status disclosure. Although 66% of survey respondents correctly identified all the ways HIV can and cannot be transmitted, there was less certainty about the life expectancy of people living with HIV (19% responded incorrectly), progression of HIV to AIDS (21% responded incorrectly) and mother–baby HIV transmission (42% responded incorrectly). The majority of interviewees had heard of U=U and PrEP but were unable to describe what they meant.

### Interpersonal level

#### Social networks

Concern about being seen attending sexual health clinics by other community members was common among interviewees. The closely connected nature of local African and Caribbean social networks intensified fears of status disclosure and negative views about HIV, which could lead to social exclusion.

#### Generational differences

Interview participants highlighted generational differences, whereby conversations about sexual health and HIV are ‘taboo’ among older people. This reduced the likelihood of open discussions about sexual health within extended family settings.

### Community level

#### HIV stigma

Fear around breaches of confidentiality by healthcare staff, leading to perceived and experienced stigma, was a major barrier to testing described by interview participants. Judgemental attitudes from healthcare staff to their sexual behaviours exacerbated stigma concerns, with participants recalling feeling ‘disgusting’. Survey respondents indicated low stigma when asked about their views about people living with HIV (such as finding out your neighbour or family member was living with HIV). By contrast, they indicated high HIV stigma when asked about feeling comfortable having a sexual relationship with someone living with HIV. This was consistent with the interview findings.

#### Taboo

Interview participants described discussing sexual health or HIV as taboo. They indicated that homophobia and moralistic attitudes linking HIV to homosexuality and sexual promiscuity contributed to this. These taboos exacerbated stigma and rendered HIV a non-topic in many ACH social circles. This reinforced cultural barriers to HIV testing, increasing a reluctance to learn one’s HIV status.

### Organisational level

#### HIV testing experiences and access inequities

Histories of insensitive, unequal treatment and racist discrimination by general health services contributed to avoidance of sexual health clinics for some interview participants. Inadequate or underfunded services targeting minoritised communities exacerbated barriers to accessible, acceptable testing options. This engendered a shared perception that ‘anyone outside of it (the community) cannot help us’ in addressing community issues.

#### Systems and services

Interviewees highlighted systemic and service-related barriers which hindered HIV testing. This included difficulty getting an appointment or accessing self-testing kits. Unclear instructions on using home testing kits and significant delays receiving results further prevented or discouraged testing.

### Theme 2: facilitators of HIV testing

#### Individual level

##### Personal motivators

Interview participants described personal motivations that drove their decisions to test. These included entering new relationships, first-hand experience of caring for patients with AIDS, pregnancy desires and having new symptoms. Some viewed testing as responsible health-promoting behaviour, or regular habit.

### Community level

#### Community attitudes

The need to dispel misconceptions about HIV was emphasised by interview participants. Updating general knowledge about advances in HIV prevention and testing could decrease stigma and encourage HIV testing. This could be achieved via targeted health events and campaigns. Having attended a CAB targeted health event, more than half of survey respondents now knew what U=U (72%) and PrEP (67%) were (a further 20% said they already had this knowledge before attending the event). The value in recruiting influencers and community leaders to champion and normalise sexual health testing was also emphasised.

#### Culturally targeted services and messaging

The CAB dedicated ACH clinics and barbershop sexual health outreach were praised for providing safe spaces to discuss sexual health and access to HIV testing. Participants appreciated these initiatives ‘coming to the people’ by being embedded within community spaces. Interview participants described tailored health messaging as vital to challenge the association of HIV testing solely with illness. They felt that normalising sexual health testing as part of a healthy lifestyle could be helpful in encouraging greater engagement.

### Organisational level

#### Improving sexual health services and access to testing

Interview participants felt that staff representation (someone who ‘looks like me’) was key to help build trust with healthcare. This included hiring more black and minority ethnic staff and ensuring cultural competence training for all staff. It was felt that this could help to address discriminatory attitudes and create a more accessible and welcoming testing environment. Participants suggested diverse representation in posters and leaflets would further promote an inclusive healthcare environment

#### Enhanced access to testing

To accommodate community needs, interview participants advocated for extended or flexible hours, walk-in appointments without referrals and mobile testing units providing decentralised care in trusted community locations. Improving convenience and privacy of testing options would also facilitate uptake. Participants liked the anonymous home testing option of self-tests but wanted more accessible instructions on how to access and use these kits.

### Policy level

#### Funding and resource allocation

Participants called for increased investments to bring testing into community spaces through outreach programmes and mobile units, to reach people who do not normally access health services. Enhancing funding would also ensure extended clinical hours, continuity of services and hiring more staff who represent the diversity of the local population.

## Discussion

The findings identify factors that hinder or promote HIV testing among ACH communities. At the individual level, there is a need to reduce misconceptions and update knowledge about advances in HIV treatment and prevention. At the interpersonal and community levels, findings suggest that peer advocates may help to normalise testing and address ‘taboo’ around sexual health. At the service level, findings highlight the need to improve access to testing in both service and community settings. Improving representation and cultural competence among service providers is crucial, given mistrust rooted in racism and negative healthcare experiences. Findings highlight that at a policy level there is an urgent need for greater investment, to achieve the factors identified at individual, community and service levels.

The individual-level barriers identified align with previous research highlighting knowledge gaps as a significant barrier to HIV testing among migrant populations in high-income countries.^2^ The current study adds nuance by revealing that even when general HIV transmission knowledge is good, significant gaps remain in understanding about modern HIV treatment and prevention methods, like U=U (Undetectable=Untransmittable) and PrEP (pre-exposure prophylaxis). This finding is important because knowledge of these advancements has the potential to significantly reduce HIV stigma, a barrier to testing. Our survey results showed high levels of stigma about dating someone living with HIV. Understanding U=U challenges the outdated notion of HIV as a ‘death sentence’ by demonstrating that people living with HIV who maintain an undetectable viral load can live long, healthy lives and cannot sexually transmit the virus. Similarly, awareness of PrEP can shift perceptions about HIV prevention and reduce fear associated with potential exposure. This knowledge can challenge internalised stigma, based on negative cultural views of HIV, which makes testing shameful and learning one’s HIV status anxiety-provoking.

Our findings reflect ongoing concerns of racially minoritised populations about HIV stigma, confidentiality, which can impact on testing uptake and frequency.^29 30^ While self-sampling kits were valued for privacy, our participants reported unclear instructions and doubts about the reliability of tests, echoing prior research.^31^ Our findings demonstrate the need for interventions to be co-produced with racially minoritised communities to ensure they are acceptable and accessible as previous researchers have suggested in relation to increasing HIV risk perception and PrEP awareness among black African and Caribbean women, to increase uptake.^32^ Our findings also demonstrated the value of community-based outreach in trusted spaces like barbershops to support increasing HIV knowledge, which previous researchers have found acceptable.^33^ These insights underscore the value of co-produced and co-delivered interventions to reduce HIV stigma, normalise testing and PrEP use. Interpersonal and community barriers included negative familial and community attitudes, increasing reluctance to attend testing clinics due to confidentiality concerns and fear of social judgement. These findings align with research on barriers to HIV testing among migrant black Africans in Western Europe, which also emphasised stigma and confidentiality issues.^4^ Interpersonal and community facilitators underscore the need for targeted education on HIV prevention and treatment, ideally supported by trusted community members. Updated information combined with respected role models can help normalise HIV testing and reduce stigma—a strategy proven effective in other studies.^34^

Negative experiences within healthcare, racism and mistrust of services were key organisational barriers. Feeling under-represented within the testing environment was a particular barrier to engagement, as were challenges in getting appointments. These findings build on previous work highlighting the impact of discrimination and access issues on HIV testing uptake.^8^ Our study contributes new insights into how culturally targeted services and community outreach can act as facilitators at this level. Participants praised initiatives like the CAB dedicated testing and PrEP clinics and barbershop outreach for providing safe spaces and ‘coming to the people’. This approach directly addresses the HIV Action Plan’s recommendation for culturally sensitive community testing.^35^ The success of CAB’s community-led approach demonstrates the effectiveness of engaging African and Caribbean communities in the design and implementation of HIV testing initiatives.

Culturally targeted services where staff and clinical resources represent people from minoritised communities, can ensure people feel comfortable and safe to test. Extended options such as walk-in clinics, longer opening hours or home self-test kits may further increase engagement. Proposed strategies like provider cultural competence training, diversifying clinical staff and outreach through trusted community channels have been effective when implemented elsewhere.^34^

The current findings did not yield extensive information on policy-level factors, but underfunding was a barrier to testing. Increased funding directed at community-based approaches aligns with recommendations from the HIV Commission’s final report^9^ on the need for sustained investment in community-led HIV prevention and testing initiatives.

The study identifies knowledge gaps, cultural stigma, systemic racism and limited access to healthcare as key barriers to HIV testing. Addressing these barriers requires a comprehensive, multilevel approach. CAB’s model—integrating community education, culturally sensitive testing and community-led initiatives—provides an effective framework for reducing these inequities. This approach supports the 2021 HIV Action Plan’s goals to address HIV stigma, promote culturally tailored testing and empower community-driven solutions for African and Caribbean communities.^35^ The findings of the full CAB evaluation will be reported separately.

### Strengths and limitations

Data was collected in a single UK city (Bristol), and therefore findings should be interpreted in light of this. The study benefitted from a diverse sample in terms of age, gender, ethnicity, migrant status and HIV testing history. Although only a minority of respondents reported a minoritised sexuality/gender, limiting the scope for intersectionality in relation to testing behaviours. The participation of community researchers from the ACH community to conduct the interviews increased trust in the research process. By prioritising the voices of ACH communities, this study provides valuable context-specific information to inform targeted local strategies to increase HIV testing access and uptake.

## Conclusion

This study identifies factors impacting HIV testing in ACH communities, particularly stigma, knowledge gaps and systemic barriers limiting uptake. However, culturally tailored services and community-based outreach show promise in increasing testing rates and reducing late diagnoses. Aligning these approaches with national health recommendations offers an opportunity to improve health outcomes and support the UK’s HIV reduction goals.

Future research should examine age, gender and identity-specific barriers, ideally through longitudinal studies assessing the lasting impact of culturally tailored interventions. Health services should prioritise culturally relevant outreach, visible representation within healthcare teams and co-produced service improvements to build trust and engagement in minoritised communities. Training in cultural competence for sexual health providers remains essential to addressing mistrust and facilitating routine HIV testing.

## Supplementary material

10.1136/sextrans-2025-056491 online supplemental file 1

10.1136/sextrans-2025-056491 online supplemental file 2

## Data Availability

All data relevant to the study are included in the article or uploaded as supplementary information.
