# Supplementary material for: Barriers and facilitators to HIV testing among African and Caribbean heritage communities: a mixed methods study
Source: Sex Transm Infect. 2025 May 13;102(1):e056491. doi: 10.1136/sextrans-2025-056491 (PMC12911657; doi:10.1136/sextrans-2025-056491)
Supplement: online supplemental file 1 [file sextrans-102-1-s001.docx]

**CAB online evaluation survey**

**The Common Ambition Bristol project**

Common Ambition Bristol (CAB) is a community powered project working with the African and Caribbean heritage communities in Bristol to tackle HIV.

You have been invited to take part in this survey because you attended a CAB event. The survey takes about 10 minutes to complete and will help improve sexual health services for African and Caribbean heritage communities in Bristol.

**Prize Draw**

If you would like to be entered into the prize draw to win £100, you can provide your contact details at the end of the survey

**What is the survey about?**

The survey includes questions about the CAB event, sexual health testing and what you think about HIV.

**Do I have to take part?**

No, taking part is voluntary and you can stop at any time.

**Is the survey confidential?**

Yes, totally confidential, and you don’t need to give your name to answer the survey.

**How will the results of the project be used?**

Anonymous data from this survey will be published and used locally and nationally to help improve sexual health services. No names or identifying details will be reported.

**Who is organising and funding the project?**

This survey is being organised by researchers from the University of Bristol. The project is funded by The Health Foundation charity.

Please answer the following question if you wish to take part in the survey.

**I agree to my survey answers being used for research**

ÿ Yes /

ÿ No - *end of questionnaire thank you for your time*

**A: Questions about You:**

**A1 Please enter your age in the box below.**

If under 18: *end of questionnaire thank you for your time*

If 18 or over: go to (A2)

**A2. Are you…?**

1. Male /
2. Female
3. Non-Binary
4. Other (please state)
5. Prefer not to say

**A3. Is your gender identity different from the gender you were given at birth?**

1. Yes
2. No /
3. Prefer not to say

**A4. How would you describe your ethnicity?**

Black /African/ Caribbean/Black British (expand to)

- African /
- Caribbean
- Any other Black/African/Caribbean background, write in

Mixed/multiple ethnic groups (expand to)

ÿ White and Black Caribbean

ÿ White and Black African

ÿ White and Asian

ÿ Any other mixed/multiple ethnic background, write in

Asian/Asian British (expand to)

ÿ Indian

ÿ Pakistani

ÿ Bangladeshi

ÿ Chinese

ÿ Any other Asian background, write in

White (expand to)

- - English / Welsh / Scottish / Northern Irish / British
  - Irish
  - Gypsy or Irish traveller
  - any other white, write in

Other ethnic group (expand to)

ÿ Arab

ÿ Any other ethnic group, write in

ÿ F Prefer not to say

**A5 What is your religion?**

1. None
2. Christian /
3. Muslim
4. Hindu
5. Jew
6. Sikh
7. Buddhist
8. Other (please state)
9. Prefer not to say

**A6 What is the highest educational level that you have achieved so far?**

1. No formal education
2. Primary school
3. Secondary school, GCSE, A-level, NVQ levels 1 to 3, etc.
4. University degree or equivalent professional qualification, NVQ level 4, etc.
5. Higher university degree, masters, PhD, NVQ level 5, etc. /
6. Still in full time education
7. Not sure
8. Prefer not to answer

**A7. How would you describe your current relationship status?**

1. Single
2. Separated / divorced / dissolved civil partnership
3. Widowed /
4. Married / in a civil partnership
5. In a relationship but **not** married / in a civil partnership
6. Prefer not to say

**A8. Do you have sex with?** (pick as many that apply)

1. Men
2. Women
3. Men and women
4. People who are non-binary
5. None / celibate
6. Prefer not to say

**B: What did you think about the CAB event**

**B1: Do you**

|  |  | Strongly agree | Agree | Disagree | Strongly disagree |
| --- | --- | --- | --- | --- | --- |
| **a** | **understand what Common Ambition Bristol (CAB) is aiming to do** |  |  |  |  |
| **b** | **feel that CAB speaks to your community well** |  |  |  |  |
| **c** | **Would you recommend a CAB event to friends or family** |  |  |  |  |

**B2: After the CAB event do you now know**

|  |  | I knew this already | Strongly agree | Agree | Disagree | Strongly disagree |
| --- | --- | --- | --- | --- | --- | --- |
| **a** | **where to find information on HIV?** |  |  |  |  |  |
| **b** | **how HIV can be passed from person to person?** |  |  |  |  |  |
| **c** | **how to get a HIV test in Bristol?** |  |  |  |  |  |
| **d** | **what PrEP is?** |  |  |  |  |  |
| **e** | **what U=U (Undetectable = Untransmittable) means?** |  |  |  |  |  |

B3 Do you know where to find the CAB website? Yes / No

**Please tell us briefly, in your own words:**

**B4. What did you like about the CAB event?**

|  |
| --- |

**B5. What could have improved this CAB event?**

|  |
| --- |

**B5. How could sexual health services be improved for African and Caribbean heritage communities in Bristol?**

|  |
| --- |

**C: HIV testing**

**C1.** **when was your last HIV test?**

1. Within the last 3 months
2. Between 3 and 12 months ago
3. Between 1 and 5 years ago /
4. More than 5 years ago
5. I have never been tested for HIV

*If e go to C1a*

**C1a Why have you never been tested for HIV?** ( one option)

1. I would rather not know my HIV status
2. I don’t think I’m at risk of having HIV
3. It’s not important to me to know my HIV status
4. I don’t know where to get tested
5. I didn't know HIV tests were free on the NHS
6. I am worried about being treated differently if I have HIV
7. It would cause problems in my relationship
8. I am too worried that I might have HIV
9. I'm afraid of needles
10. I have had a bad experience in sexual health service before
11. Other (please state)

*Go to C3*

**C2 How did you take the HIV test:**

1. In person at the GP
2. In person at a sexual health clinic
3. In person at a hospital
4. In person at a local charity or community group
5. In person at a pharmacy
6. Buying a test at a pharmacy and taking it home
7. Ordering a free NHS test online and seeing the result in a few minutes
8. Ordering a free NHS test online and sending sample to a lab to get the result
9. Other (please state)

**C3. How likely are you to go for an HIV test in the next 3 months?**

1. Very likely
2. Likely
3. Unlikely
4. Very unlikely

**C4. How would you prefer to test for HIV in the future?** (tick all from this list that apply)

1. In person at the GP
2. In person at a sexual health clinic
3. In person at a hospital
4. In person at a local charity or community group
5. In person at a pharmacy
6. Buying a test at a pharmacy and taking it home
7. Ordering a free NHS test online and seeing the result in a few minutes
8. Ordering a free NHS test online and sending sample to a lab to get the result
9. Other (please state)

**C5. Do you personally know someone who is living with HIV (has received a positive HIV test result)?**

- 1. Yes – a family member or friend
  2. Yes – a current or previous partner
  3. Yes – someone I don’t know very well, e.g. an acquaintance
  4. No
  5. I am living with HIV
  6. Don’t know
  7. Prefer not to say

**D: What do you know about HIV**

**D1. In which of the following ways, do you think HIV can be passed from person to person? (pick as many as you like)**

- 1. Kissing someone
  2. Sex without a condom between two men
  3. Oral sex without a condom or dental dam
  4. Sharing a glass
  5. From a toothbrush
  6. Spitting
  7. Biting
  8. Sex without a condom between a man and a woman
  9. A blood transfusion in the UK
  10. From a toilet seat
  11. By sharing needles or syringes
  12. Coughing or sneezing
  13. By standing on a used needle
  14. Don't know
  15. None of these

**D2. Which of these statements do you feel are true or false?**

|  |  | Certain the statement is true | Feel that the statement is true | Feel that the statement is false | Certain that the statement is false | Don't know |
| --- | --- | --- | --- | --- | --- | --- |
| **a** | Most people who have HIV in the UK will die within 5-10 years |  |  |  |  |  |
| **b** | A baby born to a mother living with HIV will also have HIV |  |  |  |  |  |
| **c** | There is zero risk of someone who is taking effective HIV treatment passing on HIV through sex |  |  |  |  |  |
| **d** | There is medicine people can take that will stop them acquiring HIV |  |  |  |  |  |
| **e** | People with HIV will always go on to develop AIDS |  |  |  |  |  |

**D3. Please read the statements and slide the button to if you agree or disagree with it.**

|  |  | Strongly agree | Strongly disagree |
| --- | --- | --- | --- |
| **a** | If I found out my neighbour was living with HIV it would not have a negative impact on my relationship with them |  |  |
| **b** | I would feel comfortable having a sexual relationship with someone living with HIV |  |  |
| **c** | If someone in my family told me that they were living with HIV, it would not have a negative impact on my relationship with them |  |  |
| **d** | My employer should have to tell me if one of my work colleagues is living with HIV |  |  |
| **e** | I don’t have sympathy for some people living with HIV because of how they got it |  |  |

**E: Could you take part in a short interview?**

The Common Ambition Bristol team would like people who have attended their event to take part in a short interview (up to 30 mins) to understand more about how sexual health services can be improved for African and Caribbean heritage communities in Bristol.

The confidential interview can be in person or by phone or online (Zoom) and you will be offered a £20 shopping voucher to thank you for your time.

**E1. I would be happy for the Common Ambition Bristol team to contact me about an interview**

ÿ Yes

ÿ No

**E2. Please provide your contact details – name and telephone number or email address**

**F: Prize Draw**

**If you would like to be entered into the prize draw to win £100, please provide your contact details – name and telephone number or email address**

**Submit your answers**

**Thank you for taking the time to complete the survey**

**HIV information**

**What is HIV?** HIV stands for Human Immunodeficiency Virus. HIV prevents the immune system from working properly. Although medication is available for someone who has HIV, they will have the virus for the rest of their life.

**How do you get HIV?** The HIV virus is spread through blood and some other bodily fluids such as blood, semen, vaginal fluid and breast milk. So it can be passed from person to person through anal or vaginal sex, through breastfeeding, or when giving birth, and through sharing needles to take drugs.

**You cannot catch HIV** from shaking hands, sharing toilets, cuddling, kissing, coughing, sneezing or spitting.

**You cannot catch HIV** from someone who is living with HIV if they are regularly taking medication for HIV which is working This is known as **U=U (Undetectable = Untransmittable).**

**PrEP** (Pre-Exposure Prophylaxis) is a pill taken by HIV-negative people every day or before and after sex that reduces the risk of getting HIV. PrEP is available for free on the NHS from your local sexual health clinic.

**PEPSE** (Post-Exposure Prophylaxis following Sexual Exposure) is a pill that can be taken if you have had sex with someone who has or might have HIV, to stop you getting HIV so long as you go to a sexual health clinic or emergency department within 72 hours of the sex.

**Free NHS HIV and STI testing kits** can be ordered online from Unity Sexual Health <https://www.unitysexualhealth.co.uk/request-postal-kit-using-online-account/>

**If you would like to find out more information about Common Ambition Bristol, sexual health, local sexual health services and PrEP, we recommend that you use the following websites:**

**Common Ambition Bristol –** <http://commonambitionbristol.org.uk/>

**Unity Sexual Health:** A free and confidential NHS sexual health service across Bristol, North Somerset and South Gloucestershire: <https://www.unitysexualhealth.co.uk/>

**Brigstowe:** Bristol charity for people living with HIV: <https://www.brigstowe.org/>

**Terrence Higgins Trust:** HIV charity working to improving the nation’s sexual health: <http://www.tht.org.uk/sexual-health>

**Prepster:** Information about PrEP: <http://prepster.info/>
