# Supplementary material for: Barriers and facilitators to HIV testing among African and Caribbean heritage communities: a mixed methods study
Source: Sex Transm Infect. 2025 May 13;102(1):e056491. doi: 10.1136/sextrans-2025-056491 (PMC12911657; doi:10.1136/sextrans-2025-056491)
Supplement: online supplemental file 2 [file sextrans-102-1-s002.docx]

**CAB Interview Topic Guide**

**INTRODUCTION:** *In this interview I would like to ask you your views about HIV; how it is viewed and talked about in the African and Caribbean communities;* *I would also like to talk to you about testing for HIV and ask your views about how sexual health services can be improved.*

**DEMOGRAPHICS:** Can you tell me your:

- Age
- Ethnicity
- How long you have lived in Bristol
- Who you live with
- What you do

**SEXUAL HEALTH CONVERSATIONS and STIGMA**`

- Have you spoken about HIV with your friends / family / community?
- If no, why do you think it’s not spoken about?
- If yes, how is it talked about?
- How do you think people living with HIV are viewed in the community? *(explore any stigma towards people living with HIV)* Probe: *can you tell me more about this / give an example*
- Do you have any particular views about people who are living with HIV?
- How likely would you talk about HIV to friends or sexual partners?
- If you met someone living with HIV would it influence if you would choose them as a friend or sexual partner? Why?
- How much did you know about HIV? Probe*: ways that HIV can be passed from person to person? / ways to prevent HIV / ways to treat HIV?*
- Have you heard of PreP? *(PrEP is a pill you can take before and after sex which can prevent you getting HIV)*
- Have you heard of U=U? *(undetectable = untransmissible, that someone living with HIV on effective medication who have undetectable levels of the virus in their blood are unable to transmit the virus to other during sex)*

**CAB preamble:** *The team from Common Ambition Bristol (CAB) have been visiting local shops and barbers to talk about HIV and sexual health. They want to encourage people in the community to talk about these issues and to get tested.*

- Have you had any conversations about sexual health, HIV or testing in a local shop / salon / barbers?

**IF YES:**

*Now I would like to ask you about the things you discussed in these conversations*

- How did this conversation come about? Where? when? Who was involved?
- What kinds of things did you discuss? *(testing, HIV info)*
- Is there anything new about HIV or sexual health that you found out?
- Did it change any of your views and understanding about HIV? If so, in what ways? *Probe: the ways that HIV can be passed from person to person / ways to prevent HIV / ways to treat HIV?*
- Did this conversation change your views on HIV?
- Have your views about people who have HIV changed in any ways? If so how?
- How likely is it now that you would talk about HIV with friends or sexual partners?
- Did you find out about how or where to test for HIV?
- Has it changed your view on getting tested for HIV?
- What did you think about having this kind of conversation in a local / shop / salon / barber?
- How could of it been improved?

**IF NO**:

- Were you aware of other people talking about these issues in X shop / salon / barber ?
- What do you think in general about these issues being discussed in your community ?How would you feel If your barber or shop owner did start a conversation about sexual health or HIV?
- Would you feel comfortable with this? Why / why not?
- Do you think it is important that conversations about sexual health and HIV are raised in your community? Why/ Why not?

**ASK ALL;**

- What do you think is the best way to share information about sexual health and HIV within the local community? *Probe: giving information leaflets / starting conversations / any other ways?*
- Are there sources of information would you trust in relation to HIV information? (*eg. NHS, GP, community groups, particular webpages)*
- Before this interview, had you heard about Common Ambition Bristol? How? do you trust CAB as a way to find out about HIV? Why/why not?
- Have you visited the CAB website? If yes, how does this help to address HIV stigma ?
- What information about HIV needs to be known to help address or change HIV stigma in the community?
- What information needs to be known to encourage people to test for HIV?

**TESTING**: *Now I would like to ask you about testing for sexual health and HIV*

- Is HIV testing something your friends talk about?
- Do you feel you have been at risk of getting HIV? Why/why not?
- Have you ever had an HIV test?

**If YES:**

What prompted you to have a HIV test? *(regular check-up, new partner felt at risk)*

how did you feel about testing? *(positive / scared / better off knowing status)*

- How often do you normally test for HIV? If more than once - how regularly do you normally have a sexual health test?
- What things influence how regularly you test? (*eg. when more at risk of sexual infections, change of relationship status, signs and symptoms, recommendations?)*
- How do you normally receive a HIV test ? Eg. *Unity clinic, going to GP or online/postal testing?*
- Why did you choose this method *(e.g. other methods not known, convenience, speed of results, anonymity)?*

**IF NEVER TESTED** (explore reasons)

- - If you wanted to test would you know how or where to go?
  - what has stopped you from testing? (e.g. not seen as a priority, not feel they are at risk, worried about test results, testing not convenient location)
  - how could these be overcome ?
  - what would encourage you to test?

Since this discussion / at the moment how likely are you to take a test?

- Where / how would you go to test? (clinic, vending machine, postal test) Why?
- How likely are you to go to Unity / a clinic? Why/ why not?

**SEXUAL HEALTH SERVICES**: *Finally, I would like to ask what you think about HIV services in Bristol*

- What might prevent people from African and Caribbean heritage communities in Bristol accessing sexual health clinic for HIV testing?
- Do you know that free NHS postal kits are available for STI test? How do you feel about these? What do you think about UNITY (the central clinic by castle park)?
- How could sexual health services be improved for African and Caribbean heritage communities in Bristol?
- Have you heard about the dedicated clinic for African and Heritage communities at Charlotte Keel ? what is your view on having a dedicated clinic?
- Given what we have already discussed, is there anything else that you want to talk about?

Thank participant and remind them that their views have been useful

Give or send them the further resources sheet

If they want further information direct them to the CAB website:

[Common Ambition Bristol | Improving Sexual Health](https://commonambitionbristol.org.uk/)

End interview
